# Progesterone receptor membrane component 1 and its role in ovarian follicle growth

**DOI:** 10.3389/fnins.2013.00099

**Published:** 2013-06-13

**Authors:** John J. Peluso

**Affiliations:** ^1^Department of Cell Biology, University of Connecticut Health Center FarmingtonCT, USA; ^2^Department of Obstetrics and Gynecology, University of Connecticut Health Center FarmingtonCT, USA

**Keywords:** progesterone, ovary, mitosis, apoptosis, progesterone receptor membrane component 1

## Abstract

Progesterone (P4) is synthesized in the ovary and acts directly on granulosa cells of developing ovarian follicles to suppress their rate of mitosis and apoptosis. Granulosa cells do not express nuclear progesterone receptor (PGR) but rather progesterone receptor membrane component-1 (PGRMC1). PGRMC1 binds P4 and mediates P4's actions, as evidenced by PGRMC1 siRNA studies. PGRMC1 acts by binding plasminogen activator inhibitor 1 RNA-binding protein and regulating gene expression. Specifically, PGRMC1 suppresses some genes that promote cell death (i.e., Bad, Caspase-3, Caspase-4). P4 regulates gene expression in part by inhibiting PGRMC1 binding to Tcf/Lef transcription sites, thereby reducing Tcf/Lef transcriptional activity. Since Tcf/Lef transcription sites are located within the promoters of genes that initiate mitosis and/or apoptosis (i.e., c-jun and c-myc), P4-PGRMC1 mediated suppression of these Tcf/Lef regulated genes could account for P4's actions. PGRMC1 expression is also altered in women with polycystic ovarian syndrome, premature ovarian failure and infertility. Collectively, these observations support a role for PGRMC1 in regulating human ovarian follicle development.

## Introduction

Progesterone receptor membrane component 1 (PGRMC1) plays a clinically important role in regulating ovarian function as demonstrated by the fact that PGRMC1 levels are reduced in some women with polycystic ovarian syndrome (Schuster et al., 2010) or premature ovarian failure (Mansouri et al., 2008; Schuster et al., 2010). In contrast PGRMC1 over expression is associated with impaired follicular development in women undergoing gonadotropin-induced ovulation and *in vitro* fertilization as part of their infertility treatment (Elassar et al., 2012). In infertile patients, the elevated levels of PGRMC1 were detected within ovarian (granulosa/luteal) cells harvested at the time of oocyte (egg) retrieval. Thus, the change in PGRMC1 expression directly reflects altered ovarian function. Moreover, PGRMC1 is highly expressed in granulosa cells of ovarian follicles of women (Engmann et al., 2006) as well as in all mammalian ovaries thus far examined including mice (Cai and Stocco, 2005), rats (Peluso et al., 2006), monkeys (Bishop et al., 2012), and cows (Kowalik and Kotwica, 2008; Luciano et al., 2011).

Interestingly, PGRMC1 is detected at the plasma membrane and cytoplasm and occasionally in the nuclei of granulosa cells of growing preantral and antral follicles (Peluso et al., 2006). This expression pattern is consistent with PGRMC1 being a mediator of progesterone's actions in granulosa cells. Specially, the ability of progesterone (P4) to slow ovarian follicular growth has been demonstrated in hypophysectomized hamsters (Moore and Greenwald, 1974), gonadotropin-primed hamsters (Kim and Greenwald, 1987) rats (Buffler and Roser, 1974), mice (Peluso et al., 1980), and monkeys (Dizerega and Hodgen, 1982). Given these findings, this mini review will focus on the experimental evidence that supports a role for P4-PGRMC1 signaling in regulating the granulosa cell functions of mitosis and apoptosis.

## PGRMC1 as a mediator of P4's actions

**Figure 1:**
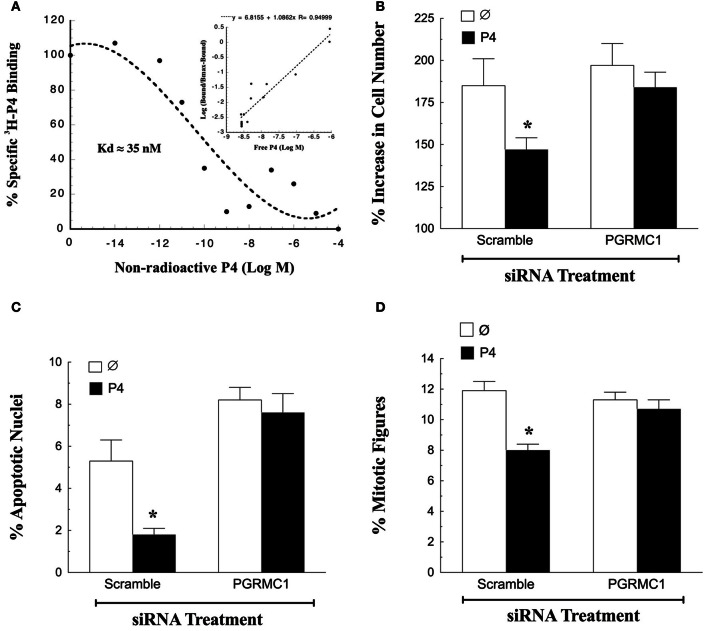
**Progesterone (P4)-PGRMC1 interactions. (A)** Shows a ligand-binding analysis of ^3^H-P4 to partially purified PGRMC1-GFP fusion protein. Specific ^3^H-P4-binding decreased with the addition of non-radioactive P4. Hill plot analysis (inset) yielded a straight line with a slope of 1.08, indicting that ^3^H-P4 specifically bound to PGRMC1-GFP in a competitive and reversible manner (Data from Figure 6 from Peluso et al., 2008). The effect of PGRMC1 siRNA on SIGC proliferation **(B)** apoptosis **(C)** and the percentage of cells in the metaphase stage of the cell cycle **(D)**. ^*^Indicates a value that is significantly different (*p* < 0.05) from scramble control (∅).

The first characteristic of a mediator of P4's action is the ability to bind P4 with high affinity. Although there are reports that PGRMC1 does not bind P4, these studies assessed P4-binding to bacterially-expressed PGRMC1 proteins (For review see Cahill, 2007). However, the bacterially-expressed PGRMC1 may not be properly folded and therefore unable to bind P4. In contrast, partially purified PGRMC1-fusion protein isolated from either spontaneously immortalized granulosa cells (SIGCs) or human granulosa/luteal cells (hGL5 cells) specifically binds P4 with high affinity (Peluso et al., 2008, 2009; Figure 1A). In addition, PGRMC1 siRNA treatment of SIGCs for 48 h depletes PGRMC1 mRNA levels to 5% of scramble control (Peluso et al., 2013). Furthermore, depleting PGRMC1 levels results in a corresponding decrease in the capacity of these cells to bind P4 (Peluso et al., 2013). Thus, these two observations provide conclusive evidence that PGRMC1 binds P4.

In addition to binding P4, PGRMC1 is an essential component in the mechanism through which P4 regulates granulosa cell function. This has been demonstrated by genetically depleting the expression of PGRMC1. Once PGRMC1 is depleted, P4 no longer inhibits apoptosis induced by serum withdrawal (Peluso et al., 2008, 2009). While serum withdrawal has been a useful model system to demonstrate a functional role for P4-PGRMC1 signaling in regulating granulosa cell survival (Peluso et al., 2008, 2009), it is limited in that SIGCs do not proliferate readily in serum-free culture. Therefore, the role of P4-PGRMC1 signaling in mitosis cannot be examined in the serum-free culture model.

To resolve this issue SIGCs were exposed to PGRMC1 siRNA in media supplemented with steroid-free serum. After 48 h, PGRMC1 levels are reduced to ≈5% of that observed after scramble siRNA treatment. In the presence of steroid-free serum, P4 suppresses the rate of mitosis and apoptosis of SIGCs exposed to scramble siRNA. In contrast depleting PGRMC1 reduces P4's ability to suppress both of these cellular functions (Figures 1B,C) (Peluso and Griffin, unpublished). Also, depleting PGRMC1 accelerates the rate at which SIGCs enter metaphase, but this increase in metaphase cells does not result in an increase in the number of cells. Rather, the metaphase cells undergo apoptosis. These studies not only confirm the functionality of the P4-PGRMC1 signaling pathway but also are consistent with the concept that P4 activation of PGRMC1 slows that rate of cell division to insure that mitosis occurs properly with fewer granulosa cells undergoing apoptosis as a result of a “mitotic catastrophe” (Peluso et al., 2012a).

**Figure 2:**
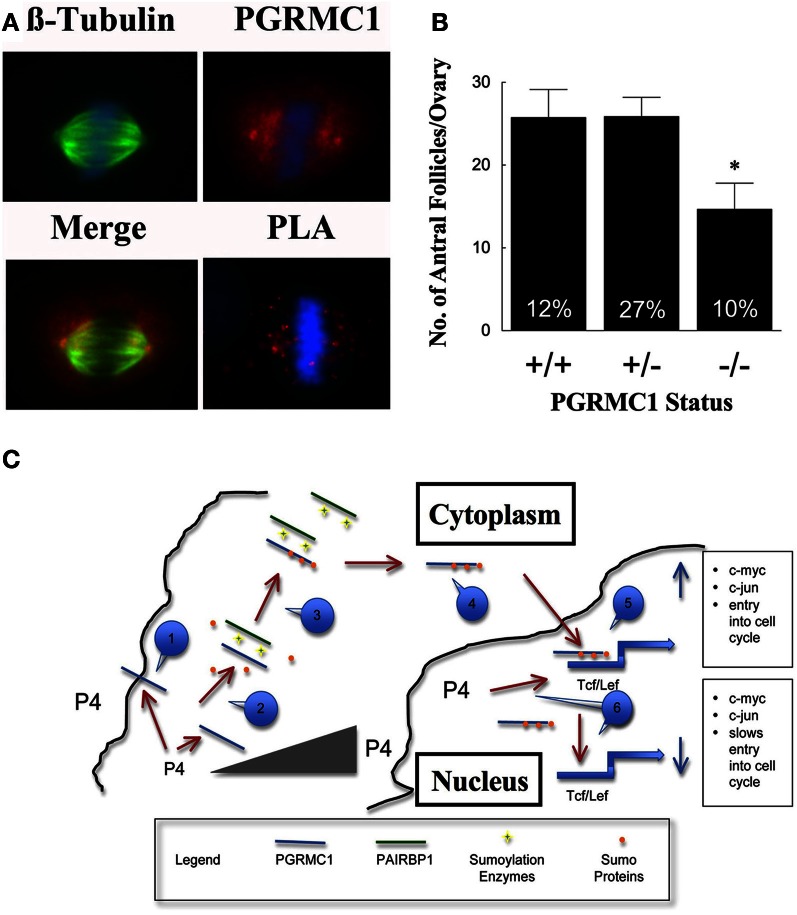
**In (A) the co-localization of β-tubulin (green) and PGRMC1 (red) in relationship to metaphase chromosomes (blue) is shown in SKOV3 cell**. Also shown is interaction between PGRMC1 and β-tubulin as revealed by *in situ* Proximity Ligation Assay (PLA). The presence of red fluorescent dots in the PLA assay indicates that two proteins are in close proximity (i.e., interacting). DNA is counterstained with DAPI (blue). Negative controls were conducted and did not show any staining. (Images are from Figures 5 to 7 from Lodde and Peluso, 2011). **(B)** Shows the number of antral follicles present in the ovaries of 22–25 day old control (+/+), heterozygous (+/−), and homozygous (−/−) PGRMC1 mice. The percentage of atretic follicles is shown at the base of each bar graph (Data from Pru and Peluso, unpublished). **(**C**)** Is a model illustrating a mechanism through which progesterone (P4) activates a PGRMC1-dependent cascade that regulates the events that initiate entry into the cell cycle. Note that a numbered blue call out box identifies the sequence of events in this mechanism and the gray triangle symbolizes increasing P4 levels within the cell. ^*^p < 0.05.

To gain insight into how P4 and PGRMC1 control mitosis, the rate at which SIGCs enter the metaphase stage of the cell cycle was determined by culturing these cells with colchicine, which arrests cells in metaphase. This study demonstrates that P4 suppresses and depletion of PGRMC1 accelerates entry into metaphase even in the presence of P4 (Figure 1D). Thus, P4 activation of PGRMC1 slows the rate of entry into the metaphase stage of mitosis. Interestingly, PGRMC1 localizes to the mitotic spindle and directly interacts with the major mitotic spindle protein, ß-tubulin, as revealed by *in situ* proximity ligation assay (Lodde and Peluso, 2011) (Figure 2A). In addition P4 increases the stability of the spindle microtubules. Since P4 slows the rate of ß-tubulin disassembly, this prolongs the duration of metaphase and extends the length of the cell cycle (Lodde and Peluso, 2011). Taken together, these results suggest that P4 activation of PGRMC1 slows the rate of mitosis at two sites by: (1) suppressing the rate of entry into the cell cycle and (2) prolonging the duration of metaphase. How P4 activation of PGRMC1 influences the rate at which the cells traverse the cell cycle at either of these sites is not known.

Based on these *in vitro* experiments, it is hypothesized that reducing the level of PGRMC1 would disrupt granulosa cell proliferation and increase apoptosis, resulting in more ovarian follicles undergoing atresia. This hypothesis would be supported if an increase in atretic (dying) follicles and/or fewer antral follicles were observed in PGRMC1 conditional knockout mice. We have recently generated mice in which PGRMC1 was depleted from granulosa cells by mating floxed PGRMC1 mice with transgenic mice in which cre recombinase enzyme is expressed under the control of the Amhr2 promoter. Using these mice a role for PGRMC1 in regulating follicle growth is supported by the observation that in immature mice with reduced PGRMC1 levels (i.e., heterozygous mice; +/−) the number of antral follicles is the same as controls but the percentage of atretic follicles is higher (Figure 2B). Depleting PGRMC1 levels as in homozygous PGRMC1 knockout mice (−/−) results in fewer antral follicles (Figure 2B) (Pru and Peluso, unpublished observations). Since the numbers of primordial, primary and preantral follicles are not reduced, this implies that PGRMC1 plays a key role in promoting the growth of ovarian follicles between the preantral and antral stages of development. The precise mechanism through which PGRMC1 promotes the development of preantral into antral follicles is not defined by likely is due in part to P4 activation of a PGRMC1 mediated cell survival pathway (Peluso et al., 2010).

These *in vitro* and *in vivo* studies together with the clinical observations imply that P4-PGRMC1 signaling plays an important role in ovarian follicle development by regulating granulosa cell proliferation and apoptosis. As indicated one site of action is at the mitotic spindle and more must be done to elucidate the details related to this site of action. Equally important is the mechanism through which P4 activation of PGRMC1 influences the rate at which granulosa cell enter into the cell cycle.

## PGRMC1 and its capacity to bind P4

It is likely that the first event in a PGRMC1-dependent signal transduction pathway is P4-binding to PGRMC1. Unfortunately, the P4-binding site within PGRMC1 has not been clearly defined. PGRMC1 is composed of a single amino acid chain of 194 amino acids. The first 20 amino acids encode the extracellular domain, amino acids 21–40 encode a transmembrane domain and amino acids 70–170 represent a cytochrome P450 b5-binding domain (Cahill, 2007). In addition there are sumoylation sites at lysine residues 136, 187, and 193 (see http://sumosp.biocuckoo.org/online.php). Chemical modification studies suggest that the progestin-binding site is within the cytoplasmic domain of PGRMC1 just distal to the transmembrane domain (Falkenstein et al., 2001). Since P4 is membrane permeable, this binding site should be relatively accessible to P4 even though it resides on the cytoplasmic side of the membrane. Interestingly, depleting any amino acid segment eliminates P4-binding, suggesting that the entire PGRMC1 molecule is required for effective P4-binding (Peluso et al., 2008). Clearly, more detailed studies regarding the structural requirements for PGRMC1 to bind P4 are needed in order to resolve this important aspect of PGRMC1's mechanism of action.

## PGRMC1 and its interaction with PAIRBP1

The sequence of molecular events that occurs once P4 binds PGRMC1 is not known. What is known is that the relationship between P4 and PGRMC1 is not a simple ligand-receptor interaction, because PGRMC1 also binds plasminogen activator inhibitor 1 mRNA-binding protein (PAIRBP1) (Peluso et al., 2005, 2008, 2013) and P4 increases this interaction (Peluso et al., 2013). Further, PAIRBP1-PGRMC1 interaction is essential for P4-PGRMC1 signaling, since depleting PAIRBP1 attenuates P4's ability to inhibit apoptosis (Peluso et al., 2013). Interestingly, depleting PAIRBP1 does not alter the expression or localization of PGRMC1 nor does it limit the ability of granulosa cells to bind P4 (Peluso et al., 2013). Thus, determining why disrupting PAIRBP1-PGRMC1 interaction is vital for P4-PGRMC1 signaling must be addressed in order to define the P4-PGRMC1 signaling cascade.

To gain insight into this issue, the amino acid sequence in PGRMC1 that promotes its interaction with PAIRBP1 was recently identified. This was accomplished by making a series of PGRMC1-GFP deletion mutations and using them in pulldown assays (Peluso et al., 2008). From these studies, it is clear that the PGRMC1 segment between amino acids 70 and 130 binds PAIRBP. In addition transfection of the 70–130 amino acid sequence of PGRMC1 into either SIGCs or primary granulosa cells disrupts the interaction between endogenous PGRMC1 and PAIRBP1 and induces cells to rapidly undergo apoptosis even in the presence of P4, PGRMC1, and PAIRBP1 (Peluso et al., 2013). This further illustrates the importance of PAIRBP1-PGRMC1 interaction.

These pulldown studies also revealed that the PGRMC1-GFP fusion protein without amino acids 131–194 binds more PAIRBP1 than the full-length PGRMC1-GFP (Peluso et al., 2008). Importantly, the 131–194 amino acid segment contains all three sumoylation sites (lysine 136, 187, and 193) (Peluso et al., 2012b). It is possible that PAIRBP1 functions as a scaffolding protein that brings PGRMC1 into close proximity to sumoylating enzymes. This concept is based on the observation that PAIRBP1, also known as CGI-55, binds enzymes that stimulate sumoylation including E3 SUMO ligases and SUMO-activating enzyme subunit 2 (Lemos and Kobarg, 2006). Under this scenario, non-sumoylated PGRMC1 would bind PAIRBP1 and become sumoylated, which would result in its released from PAIRBP1. Although this aspect of PGRMC1's mechanism of action requires intensive testing, it implies that sumoylation is an important posttranslational modification that influences the events in the P4-PGRMC1 pathway that occur after PAIRBP1-PGRMC1 interaction.

## Sumoylation of PGRMC1

To begin to validate this hypothesis, it is essential to demonstrate that PGRMC1 is sumoylated. Western blots detect PGRMC1 as a ≈22 kDa band but longer exposures often reveal several bands that are >50 kDa (Peluso et al., 2010). These higher molecular weight bands represent different forms of PGRMC1 because PGRMC1 siRNA treatment depletes these higher forms as well as the lower band (Peluso et al., 2010). Moreover, some of the higher molecular weight forms of PGRMC1 are sumoylated with SUMO1 as demonstrated by a co-immunoprecipitation (Peluso et al., 2012b). This is consistent with the finding that SUMO1 is highly expressed in the mouse ovary (Shao et al., 2004). In addition to SUMO1, there are three other SUMO family members (i.e., SUMO 2, 3, and 4) (Watts, 2007; Yan et al., 2010), which may bind to PGRMC1. While there is considerably more research required to characterize the sumoylation profile of PGRMC1, these co-immunoprecipitation studies confirm that PGRMC1 is sumoylated, thereby supporting a role for PGRMC1 sumoylation in the P4 signaling cascade.

While these co-immunoprecipitation studies are important, they are rather incomplete. Since *in silico* analysis predictes that PGRMC1 can be sumoylated at lysine residues 136, 187, and/or 193 (see http://sumosp.biocuckoo.org/online.php), an expression construct was made that encodes a PGRMC1-Flag fusion protein in which the lysine within each of the three sumoylation sites was mutated to arginine (ΔSumo-PGRMC1-Flag), thus eliminating the ability of the ΔSumo-PGRMC1-Flag fusion protein to be sumoylated. When SIGCs are transfected with either ΔSUMO-PGRMC1-Flag or wild-type PGRMC1-Flag and placed under serum-free conditions for 5 h, fewer ΔSUMO-PGRMC1-Flag transfected SIGCs undergo apoptosis (i.e., 29 ± 3%) compared to those cells that express wild type-PGRMC1-Flag (50 ± 5% apoptotic cells, *n* = 4, *p* < 0.01). Importantly, the percentage of ΔSUMO-PGRMC1-Flag transfected SIGCs that undergo apoptosis is similar to the percentage of wild type-PGRMC1-Flag transfected cells observed after treatment with P4 (i.e., 25 ± 4%; Peluso, unpublished observations).

Although it is not known whether all three sumoylation sites are functional, the fact that mutating all of the putative sumoylation sites enhances the ability of PGRMC1 to maintain SIGC viability is consistent with the concept that sumoylation plays an important role in modulating PGRMC1's actions. The precise role that sumoylation of PGRMC1 plays is under investigation. Interestingly, ΔSUMO-PGRMC1-Flag tends to be localized in the cytoplasm compared to wild-type PGRMC1-Flag, which is more equally distributed between the membrane/cytoplasmic and nuclear fractions (Peluso, unpublished observation). Given that PGRMC1-GFP without sumoylation sites binds more PAIRBP1 than wild-type PGRMC1-GFP, it is possible that the ΔSUMO-PGRMC1 remains tethered to PAIRBP1 in the cytoplasm. This putative enhanced interaction between PAIRBP1 and ΔSUMO-PGRMC1 may account for the anti-apoptotic effects of ΔSUMO-PGRMC1, since PAIRBP1-PGRMC1 interaction is essential for cell survival (Peluso et al., 2013).

In addition, sumoylation is an important posttranslational modification that often promotes the transport of a protein from cytoplasmic to the nucleus (Geiss-Friedlander and Melchior, 2007; Watts, 2007). This is consistent with the observation that the higher molecular weight forms of PGRMC1 are located in the nucleus (Peluso et al., 2010, 2012b). The nuclear localization also implies that PGRMC1 may be involved in regulating gene transcription.

## PGRMC1's nuclear localization and genomic action

Clearly, PGRMC1 sumoylation is an important event in the P4-PGRMC1 signal cascade but this signal cascade has additional components. This is evident because P4's ability to prevent apoptosis requires RNA synthesis (Peluso et al., 2010). Since PGRMC1 is present within the nucleus (Peluso et al., 2010, 2012b), it is likely that P4 activation of nuclear PGRMC1 regulates gene expression. To assess this, experiments were conducted to identify genes whose expression is dependent on PGRMC1 (Peluso et al., 2010, 2012a). Although the expression of some genes was shown to be PGRMC1-dependent (i.e., Bad, Caspase-3, Caspase-4) (Peluso et al., 2010, 2012a), these studies are difficult to assess because the expression of PGRMC1-dependent genes may not be the initial event that is triggered by P4-activated PGRMC1. Therefore, studies were conducted to identify transcription factors, whose activity was regulated by P4 activation of PGRMC1. A total of 48 transcription factors were screened and Tcf/Lef identified as a transcription factor site whose activity is suppressed by P4 (Peluso et al., 2012b).

To validate this transcription factor screen, filter and Tcf/Lef luciferase reporter assays were used and these assays confirmed that P4 decreases Tcf/Lef activity. Further, P4's ability to suppress Tcf/Lef luciferase reporter activity is PGRMC1-dependent because PGRMC1 siRNA attenuates P4's ability to suppress Tcf/Lef activity.

Interestingly, when PGRMC1 levels are increased by forcing the expression of PGRMC1-Flag fusion protein, Tcf/Lef activity is increased by 2-fold and P4 suppresses the increased Tcf/Lef luciferase reporter activity induced by the PGRMC1-Flag (Peluso et al., 2012b). Gel shift assays were also used to expand the luciferase-based estimates of Tcf/Lef activity and demonstrate that P4 decreases the amount of PGRMC1 bound to the Tcf/Lef DNA probe. Further super shift assays reveal that PGRMC1-Flag fusion protein binds to the Tcf/Lef DNA probe (Peluso, unpublished observation). The ability of PGRMC1 to either enhance or suppress Tcf/Lef activity in a P4-dependent manner is consistent with concept that PGRMC1 acts as switch to either induce or suppress Tcf/Lef dependent gene expression. This concept is further supported by the fact that Tcf/Lef-binding sites are found within the promoter region of c-myc (Wierstra and Alves, 2008) and c-jun (Mann et al., 1999), two genes that can initiate either mitosis or apoptosis (Dang and Lewis, 1997).

## A putative model of the P4-PGRMC1 signal transduction pathway and entry into the cell cycle

Based on these studies, a preliminary mechanism can be outlined that defines the P4-PGRMC1 signaling cascade in the context of ovarian follicle development (Figure 2C). This mechanism assumes that (1) the source of P4 is from either within individual granulosa cells, secreted from granulosa cells within an individual follicle or secreted from adjacent ovarian follicles or corpora lutea, (2) regardless of the source, P4 acts on PGRMC1 that is present at or near the plasma membrane and within the nucleus, and (3) P4's actions are dose-dependent. With these assumptions in mind, it is proposed that this signal transduction cascade is initiated by P4-binding to PGRMC1 at or near the plasma membrane. This binding occurs at relatively low P4 concentrations since the *K*_d_ for PGRMC1-binding to P4 is between 10 and 40 nM (Peluso et al., 2008, 2009). P4-binding likely alters the non-sumoylated form of PGRMC1 in such a way as to facilitate its interaction with PAIRBP1, which is present in cytoplasm. PAIRBP1 acts as a scaffolding protein bringing sumoylation enzymes into close proximity with PGRMC1. As a result, PGRMC1 is sumoylated and its ability to bind PAIRBP1 is reduced, thereby releasing PGRMC1 and facilitating its transport into the nucleus.

Once in the nucleus, it is proposed that PGRMC1 interacts with Tcf/Lef-binding sites that are within the promoters of early immediate genes. PGRMC1 in the presence of mitogenic stimuli enhances Tcf/Lef activity, which in turn stimulates the expression of early immediate genes (e.g., c-myc and c-jun) and subsequently induces the granulosa cell to enter the cell cycle. This mechanism is consistent with the observations that in growing follicles PGRMC1 is localized to the nucleus of about 30% of granulosa cells, which is the same as the percentage of granulosa cells that are in the S-phase of the cell cycle (Pedersen, 1970). However, as these follicles grow, they synthesize more P4 (Roy and Greenwald, 1987). The increasing levels of P4 feeds back to reduce capacity of nuclear PGRMC1 to bind these Tcf/Lef-binding sites. This would make it more difficult for mitogenic stimuli to induce the expression of genes that initiate mitosis and would ultimately slow the rate of granulosa cell proliferation. This is in agreement with the finding that as antral follicle increase in size the frequency of granulosa cells in the S-phase of the cell cycle decreases (Pedersen, 1970) and steroid synthesis increases (Roy and Greenwald, 1987; Hirshfield, 1991). How P4 alters PGRMC1 and thereby its interaction with the Tcf/Lef site is not known but may related to the phosphorylation state of PGRMC1, since phosphorylation alters the function of PGRMC1 (Neubauer et al., 2008). Thus, this proposed negative feedback system could explain in part the inverse relationship between rate of follicle growth and steroidogenesis (Hirshfield, 1991).

While this proposed mechanism is incomplete at present, it is proposed to provide a framework for future studies on P4's action in granulosa cells. Since PGRMC1 is expressed in numerous tissues as outlined in several of the reviews in this issue, it is also hoped that this putative mechanism will served as a guide to help elucidate the mechanism of PGRMC1's action in other tissues.

### Conflict of interest statement

The author was awarded a patent on non-genomic regulators of progesterone's action.
